# Deletion of epithelial HKDC1 decelerates cellular proliferation and impairs mitochondrial function of tumorous epithelial cells thereby protecting from intestinal carcinogenesis

**DOI:** 10.1002/cac2.70022

**Published:** 2025-03-20

**Authors:** Lea Järke, Saskia Weber‐Stiehl, Kensuke Shima, Karlis Arturs Moors, Jerome Genth, Fenja Amrei Schuran, Lena Best, Markus Tschurtschenthaler, Burkhardt Flemer, Silke Lüschen, Christoph Röcken, Andreas Tholey, Christoph Kaleta, Jan Rupp, Philip Rosenstiel, Felix Sommer

**Affiliations:** ^1^ Institute of Clinical Molecular Biology, Kiel University Kiel Germany; ^2^ Institute of Medical Microbiology, University of Lübeck Lübeck Germany; ^3^ Institute of Experimental Medicine, Kiel University Kiel Germany; ^4^ Translational Cancer Research and Institute of Experimental Cancer Therapy, Klinikum rechts der Isar School of Medicine and Health, Technical University of Munich Munich Germany; ^5^ Center for Translational Cancer Research (TranslaTUM), Klinikum rechts der Isar, School of Medicine and Health, Technical University of Munich Munich Germany; ^6^ Division of Translational Cancer Research German Cancer Research Center (DKFZ) and German Cancer Consortium (DKTK) Heidelberg Germany; ^7^ Department of Pathology University Hospital Schleswig‐Holstein, Campus Kiel Kiel Germany; ^8^ Infectious Disease Clinic University Hospital Schleswig‐Holstein/Campus Lübeck Lübeck Germany; ^9^ German Center for Infection Research (DZIF), Partner Site Hamburg‐Lübeck‐Borstel‐Riems Lübeck Germany

**List of Abbreviations**
ADP: Adenosine diphosphate
ANOVA: Analysis of variance
ANT2: Adenine nucleotide translocator 2
*Apc* ^Min/+^
: Adenomatous‐polyposis‐coli multiple intestinal neoplasia
ATP: Adenosine triphosphate
ATP5A1: ATP Synthase Subunit Alpha 1
CRC: Colorectal cancer
DAPI: 4′,6‐diamidino‐2‐phenylindole
Dpi: Days post infection
ECAD: E‐Cadherin
FACS: Fluorescence activated cell sorting
FKPM: Median Fragments Per Kilobase of exon per Million reads
GCK: Glucokinase
GFPT1: Glutamine‐Fructose‐6‐Phosphate Transaminase 1
GTEx: The Genotype‐Tissue Expression
HC: Healthy controls
HK: Hexokinase
HKDC1: Hexokinase domain containing 1
HPA: Human Protein Atlas
IEC: Intestinal epithelial cell
IFN‐β: Interferon beta
MPTP: Mitochondrial permeability transition pore
NSG: NOD.Cg‐Prkdc^SCID^ Il2rg^tm1Wjl^/SzJ
nTPM: normalized protein‐coding transcripts per million
OCR: Oxygen consumption rate
RNA‐seq: Ribonucleotide acid sequencing
SEM: Standard error of the mean
SLC25A5: Solute Carrier Family 25 Member 5
STRING: Search Tool for the Retrieval of Interacting Genes/Proteins
STS: Staurosporine
TCGA: The Cancer Genome Atlas
TMRM: Tetramethylrhodamine‐methyl ester
TNF: Tumor necrosis factor
TUNEL: Terminal deoxynucleotidyl transferase dUTP nick end labelling
VDAC: Voltage‐dependent anion channel
WT: Wildtype

1

A metabolic switch favoring glycolysis over aerobic oxidative phosphorylation, termed the “Warburg effect”, is a hallmark of cancer cells [1]. Hexokinase (HK) catalyzes the first and irreversible step of glycolysis, thereby limiting overall glycolytic activity. Mammals encode five HK family members: HK1‐4 and HKDC1 (HK domain containing 1). HKDC1 has an exceptionally low glucose affinity and, therefore, low hexokinase activity under physiological conditions [2], raising questions about its function. A recent study indicated that HKDC1 functions as a glucose sensor within the tumor microenvironment [3], and its dysregulated expression has been associated with chronic inflammation [4] and various cancers [5]. Notably, HKDC1 promotes tumor immune evasion during immunotherapy in hepatocellular carcinoma patients [6], and blocking HKDC1 prevents disease progression in hepatic carcinoma, T cell lymphoma, and lung adenocarcinoma [7, 8]. However, its role in colorectal cancer (CRC) remains unknown. Here, we functionally investigated the role of HKDC1 in intestinal carcinogenesis.

First, we analyzed *HKDC1* expression in the intestinal mucosa of healthy controls (HC) and CRC patients, as well as in various tumor tissues, using transcriptomic data from the human protein atlas (HPA) (https://www.proteinatlas.org) and The Cancer Genome Atlas (TCGA) (https://www.cancer.gov/ccg/research/genome‐sequencing/tcga). Across different organs, *HKDC1* showed the highest expression levels in the gastrointestinal tract (Supplementary Figure S1A), suggesting a potential role in intestinal function. Among various cancer types, *HKDC1* expression was highest in CRC, followed by pancreatic, renal, stomach, and liver cancer (Supplementary Figure S1B). *HKDC1* expression was significantly elevated in the intestinal mucosa of CRC patients compared to HC (Figure 1A) and in paired tumor versus normal tissue of the same CRC patients (Figure 1B). This overexpression appears to be more pronounced in tumor tissue, suggesting a potential association with cancer development rather than a general disease‐related effect.

**FIGURE 1:**
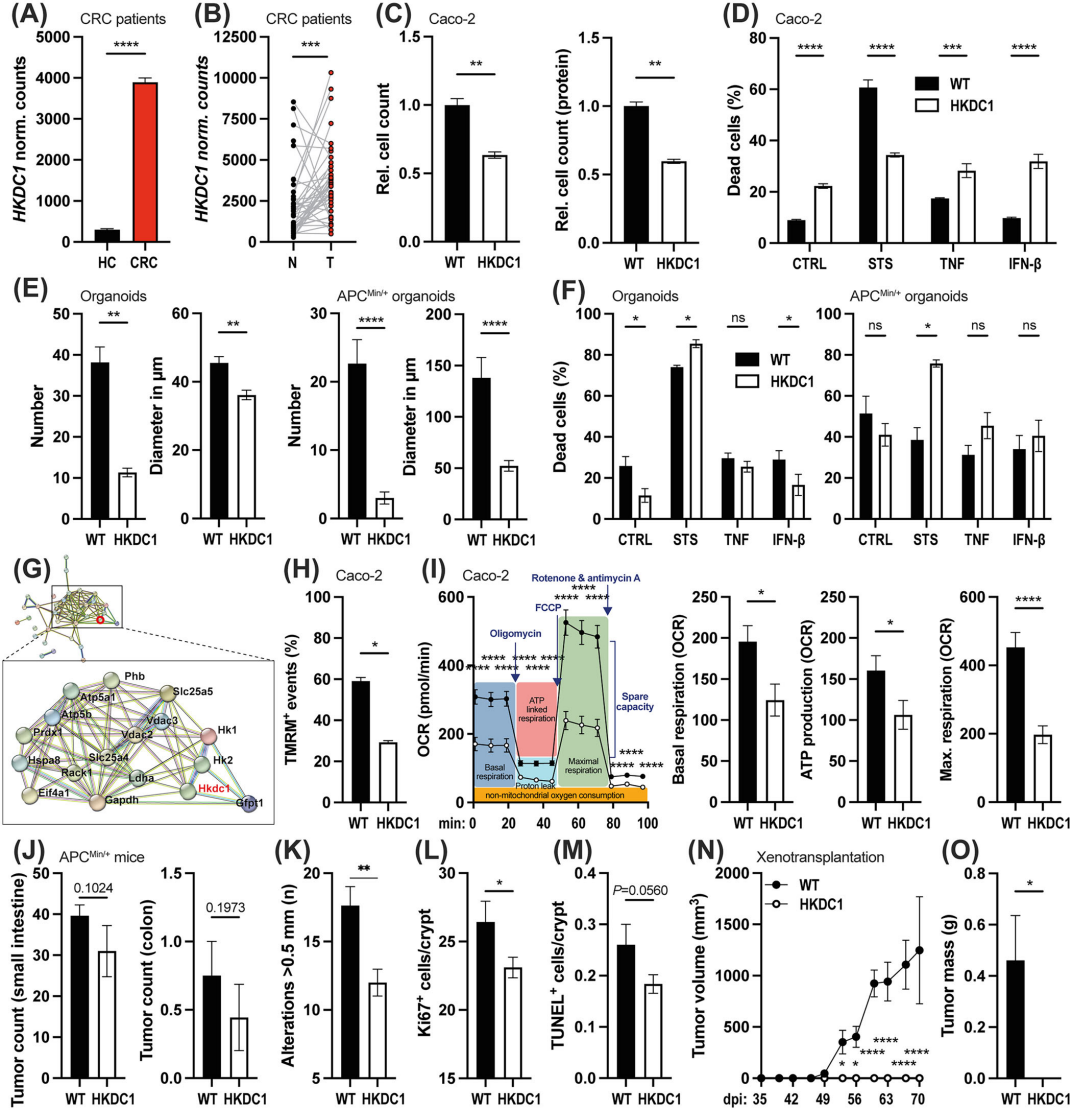
*HKDC1* expression is associated with colorectal cancer. (A) *HKDC1* expression (normalized counts) in the intestinal mucosa of healthy controls (HC) and CRC patients, retrieved from The Genotype‐Tissue Expression (GTEx, https://gtexportal.org, *n*  =  454 samples) and TCGA (*n*  =  651 samples). (B) *HKDC1* expression in paired normal (N) and tumor (T) tissues of *n*  = 41 CRC patients from TCGA. *** *P* < 0.001, Wilcoxon rank test. (C‐F) HKDC1 deletion alters proliferation and cell death. Proliferation, measured by cell count four days post‐seeding or by protein amount, was reduced in HKDC1‐deficient human Caco‐2 cells(C) (*n* =  4 ‐ 6 per group). WT and HKDC1‐deficient Caco‐2 cells were stimulated with either staurosporine(D) (STS, 10 µmol/L for Caco‐2), tumor necrosis factor (TNF, 500 ng/µl), or interferon beta (IFN‐β, 1000 U/µl) for 24 hours, and cell death was assessed through zombie staining and FACS analysis. *n* = 5 per group. HKDC1‐deficient normal and tumorigenic *Apc* ^Min/+^ organoids exhibited reduced growth, as determined by an organoid forming assay (E). The number and diameter of organoids were measured five days after seeding (*n*  =  6 ‐ 12 per genotype and group). Percentage of dead cells in normal and tumorigenic *Apc* ^Min/+^ organoids after stimulation with STS, TNF, or IFN‐β for 24 hours (F) (*n*  =  5 per group). (G‐I) HKDC1 deficiency impairs mitochondrial function. Network of HKDC1 interaction partners based on STRING analysis of proteins identified from HKDC1 immunoprecipitation of intestinal mucosa, highlighting a cluster of mitochondria‐related proteins (G). Nodes represent individual proteins. Lines indicate known (cyan = curated databases, magenta = experimentally determined) and predicted (green = gene neighborhood, red = gene fusions, blue = gene co‐occurrence) interactions. Mitochondrial phenotyping of WT and HKDC1‐deficient human Caco‐2 cells. Deletion of HKDC1 resulted in (H) reduced mitochondrial membrane potential, as measured by TMRM staining and FACS analysis, and (I) decreased oxygen consumption rate (OCR) as a measure of mitochondrial activity, determined by Seahorse Mito Stress metabolic analysis. Basal respiration, maximal respiration, and ATP production were significantly reduced in HKDC1‐deficient cells. *n*  =  9 ‐ 10 per genotype. (J‐O) Epithelial deletion of *HKDC1* ameliorates intestinal carcinogenesis. (J‐M) Sporadic intestinal tumorigenesis in the *Apc* ^Min/+^ mouse model. 20‐week‐old tumor‐bearing *Apc* ^Min/+^‐*Hkdc1* ^∆IEC^ mice were compared to WT littermate controls and analyzed for (J) tumor numbers, (K) the number of lesions > 0.5 mm, (L) proliferating (Ki67‐positive), and (M) apoptotic (TUNEL‐positive) colonic IECs. *n*  =  9 ‐ 10 per genotype. *: *P* < 0.05, Mann‐Whitney‐U‐test. (N‐O) Xenograft model. NSG mice were injected with WT and HKDC1‐deficient human Caco‐2 cells, and tumor volume (mm^3^) was monitored over time (N). At 70 days post‐injection (dpi), the experiment was terminated for ethical reasons, and total tumor mass (g) was measured (O). *n*  =  4 mice per injection group. *P < 0.05, **P < 0.01, ***P < 0.001, and ****P < 0.0001, Mann‐Whitney‐U‐test or two‐way ANOVA. All data are presented as mean ± SEM. ANOVA, Analysis of variance; Apc^Min/+^, Adenomatous‐polyposis‐coli multiple intestinal neoplasia; ATP, Adenosine triphosphate; CRC, Colorectal cancer; Dpi, Days post infection; FACS, Fluorescence activated cell sorting; FKPM, Median Fragments Per Kilobase of exon per Million reads; GTEx, The Genotype‐Tissue Expression; HC, Healthy controls; HK, Hexokinase; HKDC1, Hexokinase domain containing 1; HPA, Human Protein Atlas; IEC, Intestinal epithelial cell; IFN‐β, Interferon beta; MPTP, Mitochondrial permeability transition pore; N, normal tissue; NSG, NOD.Cg‐PrkdcSCID Il2rgtm1Wjl/SzJ; OCR, Oxygen consumption rate; SEM, Standard error of the mean; STRING, Search Tool for the Retrieval of Interacting Genes/Proteins; STS, staurosporine; T, tumor tissue; TCGA, The Cancer Genome Atlas; TMRM, Tetramethylrhodamine‐methyl ester; TNF, Tumor necrosis factor; TUNEL, Terminal deoxynucleotidyl transferase dUTP nick end labelling; VDAC, Voltage‐dependent anion channel; WT, Wildtype.

We then generated HKDC1‐deficient human (Caco‐2) and murine (CMT‐93) colonic epithelial cell lines using CRISPR/Cas9 technology, and intestinal organoids, to investigate the role of HKDC1 in proliferation and cell death susceptibility in vitro, since uncontrolled cell division and resistance to cell death are hallmarks of cancer cells. Both Caco‐2 and CMT‐93 cells express *HKDC1, HK1*, and *HK2*, but with differential expression patterns (Supplementary Figure S2). Wildtype (WT) and HKDC1‐deficient cells were seeded at equal densities, and after four days, cell growth was assessed using a colony‐forming assay or protein quantification as a molecular measure of cell number. HKDC1‐deficient cells displayed significantly reduced cell counts and protein content compared to WT cells (Figure 1C, Supplementary Figure S3A), indicating that HKDC1 contributes to cellular proliferation. To investigate whether HKDC1 affects sensitivity to cell death induction, WT and HKDC1‐deficient Caco‐2 and CMT‐93 cells were stimulated with cell death‐inducing agents, and cell viability was analyzed. Under all conditions, except for staurosporine‐treated Caco‐2 cells, HKDC1‐deficient cells showed a higher percentage of dead cells than WT controls (Figure 1D, Supplementary Figure S3B). These findings suggest that HKDC1 loss influences the cell death response in cancerous epithelial cells, potentially by affecting mitochondria‐dependent cell death. No compensatory upregulation of HK1 or HK2 was observed in HKDC1‐deficient cells (Supplementary Figure S3C), although off‐target effects of staurosporine cannot be ruled out due to its non‐selective protein kinase inhibitor activity. These findings were further validated *ex vivo* using non‐transformed intestinal epithelial organoids. HKDC1‐deficient organoids were derived from *Hkdc1* ^∆IEC^ mice (Supplementary Figure S4), which carry a *Hkdc1* deletion specifically in intestinal epithelial cells (IECs). Organoid formation assays revealed that HKDC1‐deficient organoids grew more slowly and exhibited reduced overall cell mass compared to WT organoids (Figure 1E). To assess the impact of HKDC1 loss in intestinal tumorigenesis, *Hkdc1* ^∆IEC^ mice were crossbred with tumor‐bearing *Apc* ^Min/+^ mice, a model of sporadic intestinal carcinogenesis [9]. HKDC1‐deficient *Apc* ^Min/+^ organoids, derived from intestinal tissue including tumors, also showed slower growth and reduced cell mass compared to controls (Figure 1E). Treatment with staurosporine, which triggers cell death via mitochondrial cytochrome c release, induced cell death in normal organoids regardless of HKDC1 genotype. However, while WT *Apc* ^Min/+^ tumor organoids were completely resistant to cell death, HKDC1 deletion restored cell death susceptibility (Figure 1F). Transcriptional profiling and gene ontology enrichment analysis revealed alterations in proliferation and cell death pathways, as well as metabolic and immune processes, in HKDC1‐deficient *Apc* ^Min/+^ organoids compared to WT controls (Supplementary Figure S5). Collectively, these data demonstrate that HKDC1 loss in colonic epithelial cells affects both proliferation and cell death dynamics.

Seeking a more comprehensive understanding HKDC1's role in cellular function, we performed HKDC1 immunoprecipitation. Lysates from the intestinal mucosa of *Hkdc1* ^∆IEC^ and WT mice were incubated with an HKDC1 antibody bound to Dynabeads, followed by washing, purification, and LC‐MS analysis. After excluding cytoskeletal proteins, we identified 34 candidate HKDC1 interaction partners (Supplementary Table S1). A protein interaction network analysis revealed a distinct cluster of proteins associated with the regulation of mitochondrial membrane potential and pore activity (Figure 1G). This cluster included other HK isoforms, HK1 and HK2, as well as VDAC2 (Voltage‐dependent anion channel 2), VDAC3, SLC25A5 (Solute Carrier Family 25 Member 5 or ADP/ATP Translocase 2), ATP5A1 (ATP Synthase Subunit Alpha 1), and GFPT1 (Glutamine‐Fructose‐6‐Phosphate Transaminase 1). These proteins play roles in mitochondrial metabolism and the mitochondrial permeability transition pore (MPTP), although the exact composition of the MPTP remains unclear [10]. To investigate mitochondrial membrane potential, we used MitoProbe tetramethylrhodamine‐methyl ester (TMRM). TMRM signals were consistently reduced in HKDC1‐deficient compared to WT cells, indicating a decrease in mitochondrial membrane potential in the absence of HKDC1 (Figure 1H, Supplementary Figure S6A). Additionally, Seahorse analyses were performed. Consistent with the findings from TMRM, HKDC1‐deficient cells showed significantly reduced mitochondrial respiration (Figure 1I, Supplementary Figure S6B). Basal respiration along, along with ATP‐linked and maximal respiration, was lower in HKDC1‐deficient cells, highlighting a dysfunctional mitochondrial electron transport chain. Together, these data suggest a crucial role for HKDC1 in mitochondrial function, potentially due to its association with mitochondria and MPTP‐related proteins.

To directly assess HKDC1's role in intestinal carcinogenesis in vivo, we phenotyped tumor‐bearing *Apc* ^Min/+^‐*Hkdc1* ^∆IEC^ mice. Although body and organ weights did not differ between *Apc* ^Min/+^‐*Hkdc1* ^∆IEC^ and WT mice (Supplementary Figure S7), intestinal tumor counts in *Apc* ^Min/+^‐*Hkdc1* ^∆IEC^ trended lower compared to WT mice, though this did not reach statistical significance (*P*  =  0.102 for the small intestine and *P*  =  0.197 for the colon (Figure 1J). This lack of significance may be due to technical limitations in identifying macroscopic tumors in the tissue. Further analysis showed that tumor burden, measured as the percentage of the affected area in intestinal Swiss rolls, did not differ between WT and *Apc* ^Min/+^‐*Hkdc1* ^∆IEC^ mice. However, *Apc* ^Min/+^‐*Hkdc1* ^∆IEC^ mice showed fewer lesions larger than 0.5 mm (Figure 1K), suggesting that HKDC1 may influence tumor growth rather than initiation. Supporting this, histological analyses of colon sections revealed a significant reduction in Ki67‐positive proliferating IECs in *Apc* ^Min/+^‐*Hkdc1* ^∆IEC^ mice (0.87‐fold, *P*  =  0.038) and a slight decrease in apoptotic TUNEL‐positive IECs (0.71‐fold, *P*  =  0.056) (Figure 1L‐M). To further corroborate these findings, we performed a xenograft transplantation model, where WT and HKDC1‐deficient Caco‐2 cells were subcutaneously injected into the flanks of immunocompromised NSG mice, and tumor growth was monitored over time. By 60 days post‐injection (dpi), mice transplanted with WT cells began developing visible tumors, which continued to grow until 70 dpi. In stark contrast, none of the mice transplanted with HKDC1‐deficient cells developed any detectable tumor (Figure 1N‐O). Thus, deletion of HKDC1 in human colonic epithelial cells completely abolished their tumor‐forming ability, providing full protection against cancer development. It is important to note that, despite similar physiological responses, differences exist among the models used. For example, CMT‐93 and Caco‐2 cell lines express different levels of HK family members, and the *Apc* ^Min/+^ model primarily represents small intestine‐driven tumorigenesis due to an APC mutation in the Wnt signaling pathway, whereas our xenotransplantation model used colonic epithelial cells to more closely resemble human CRC. Further investigations using additional models are needed to elucidate the underlying molecular mechanisms.

Our findings demonstrate that HKDC1 influences cancer cell proliferation, susceptibility to cell death, and ultimately intestinal carcinogenesis, potentially through interactions with mitochondrial proteins regulating membrane permeability. However, the precise molecular mechanism remains unclear. Collectively, our data highlight the significance of HKDC1 in CRC pathobiology, presenting it as a promising target for further investigation and potential therapeutic interventions. However, further studies are required to uncover the molecular mechanisms by which HKDC1 affects cellular physiology and to access the feasibility and efficacy of HKDC1‐targeted interventions for CRC.

## AUTHOR CONTRIBUTIONS

Lea Järke, Saskia Weber‐Stiehl, Kensuke Shima, Jerome Genth, and Felix Sommer designed the research. Lea Järke, Saskia Weber‐Stiehl, Kensuke Shima, Karlis Arturs Moors, Jerome Genth, Fenja Amrei Schuran, Lena Best, Markus Tschurtschenthaler, Burkhardt Flemer, Silke Lüschen, and Felix Sommer performed experiments and analyzed the data. Christoph Röcken, Andreas Tholey, Christoph Kaleta, Jan Rupp, and Philip Rosenstiel contributed critical resources. Lea Järke, Saskia Weber‐Stiehl, and Felix Sommer prepared the figures. Lea Järke and Felix Sommer obtained funding. Lea Järke, Saskia Weber‐Stiehl, and Felix Sommer co‐wrote the manuscript with critical input from all authors. All authors read and approved the final manuscript.

## CONFLICT OF INTEREST STATEMENT

Philip Rosenstiel reports stock ownership in Gerion Biotech GmbH and consulting fees from Takeda. All other authors declare no competing interests.

## FUNDING INFORMATION

This work was supported by the German Research Foundation (DFG) through the individual grant SO1141/10‐1, the Research Unit FOR5042 “miTarget‐The Microbiome as a Target in Inflammatory Bowel Diseases” (project P5), the Excellence Cluster EXS2167 “Precision Medicine in Chronic Inflammation”, an intramural grant of the medical faculty of Kiel University (grant no K126408) to Felix Sommer and ZMB Young Scientist Award 2021, category doctoral students (grant no F384430) to Lea Järke. The funding bodies had no part or influence on the design of the study and data collection, analysis, or interpretation.

## ETHICS APPROVAL AND CONSENT TO PARTICIPATE

All animal experiments were approved by the local animal safety review board of the federal ministry of Schleswig Holstein and conducted according to national and international laws and policies (approval numbers: V242‐56302/2018[100‐11/18] and IX552‐65205/2024[24‐4/24]). No human studies were conducted but only data from public databases used.

## Supporting information



Supporting information

## Data Availability

All data is either included in this manuscript or deposited on public databases. The RNA sequencing data are accessible through the European Nucleotide Archive (https://www.ebi.ac.uk/ena) under the accession number PRJEB82610.
