# Supplementary material for: Deletion of epithelial HKDC1 decelerates cellular proliferation and impairs mitochondrial function of tumorous epithelial cells thereby protecting from intestinal carcinogenesis
Source: Cancer Commun (Lond). 2025 Mar 20;45(7):722–7. doi: 10.1002/cac2.70022 (PMC12328089; doi:10.1002/cac2.70022)
Supplement: Supplementary file 1 — Supporting information [file CAC2-45-722-s001.docx]

**Supplementary Materials**

**Deletion of epithelial HKDC1 decelerates cellular proliferation and impairs mitochondrial function of tumorous epithelial cells thereby protecting from intestinal carcinogenesis**

Lea Järke^1,†^, Saskia Weber-Stiehl^1,†^, Kensuke Shima^2,#^, Karlis Arturs Moors^3,#^, Jerome Genth^3,#^, Fenja Amrei Schuran^1^, Lena Best^3^, Markus Tschurtschenthaler^4,5^, Burkhardt Flemer^1^, Silke Lüschen^6^, Christoph Röcken^6^, Andreas Tholey^3^, Christoph Kaleta^3^, Jan Rupp^2,7,8^, Philip Rosenstiel^1^, Felix Sommer^1,*^

^1^Institute of Clinical Molecular Biology, Kiel University, Kiel, Germany.

^2^Institute of Medical Microbiology, University of Lübeck, Lübeck, Germany.

^3^Institute of Experimental Medicine, Kiel University, Kiel, Germany.

^4^Center for Translational Cancer Research (TranslaTUM), Klinikum rechts der Isar, School of Medicine and Health, Technical University of Munich, Munich, Germany.

^5^Division of Translational Cancer Research, German Cancer Research Center (DKFZ) and German Cancer Consortium (DKTK), Heidelberg, Germany.

^6^Department of Pathology, University Hospital Schleswig-Holstein, Campus Kiel, Kiel, Germany.

^7^Clinic for Infectious Diseases, University of Lübeck, Lübeck, Germany.

^8^German Center for Infection Research (DZIF), Partner Site Hamburg-Lübeck-Borstel-Riems, Lübeck, Germany.

^†^Lea Järke and Saskia Weber-Stiehl contributed equally with shared first authorship.

^#^Kensuke Shima, Karlis Arturs Moors, and Jerome Genth contributed equally with shared second authorship.

^*^**Corresponding authors:**

Felix Sommer; Institute of Clinical Molecular Biology (IKMB), Christian-Albrechts-University (CAU) Kiel, Rosalind-Franklin-Str. 12, Kiel D-24105, Germany; tel: +49(0) 431/500-15146; email: [f.sommer@ikmb.uni-kiel.de](mailto:f.sommer@ikmb.uni-kiel.de).

**Supplementary Materials and Methods**

**Analyses of HKDC1 expression in human CRC patients**

RNA-seq data from normal and tumor tissues of human cancer patients were retrieved from multiple public databases: the Human Protein Atlas (HPA, [www.proteinatlas.org](http://www.proteinatlas.org/)) [1], The Cancer Genome Atlas (TCGA, <https://www.cancer.gov/ccg/research/genome-sequencing/tcga>) [2,3], and The Genotype-Tissue Expression (GTEx, available at <https://gtexportal.org/home>) [4]. Tissue RNA expression data from HPA were reported as nTPM (normalized protein-coding transcripts per million), representing the mean values of individual samples from each tissue. RNA-seq data from 17 cancer types (HPA) were reported as median fragments per kilobase of exon per million reads (FPKM). RNA-seq data from healthy controls and CRC patients (GTEx and TCGA), as well as from paired normal and tumor tissues of CRC patients (TCGA), were reported as normalized counts.

**Mice**

All animal experiments were approved by the local animal safety review board of the federal ministry of Schleswig Holstein and conducted in accordance with national and international regulations (approval numbers V242-56302/2018[100-11/18] and IX552-65205/2024[24-4/24]). Mice were provided with autoclaved water and food ad libitum and maintained in a 12-hour light-dark cycle under standardized conditions (21 °C ± 2 °C with 60 % ± 5 % humidity) at the Central Animal Facility (ZTH) of the University Hospital Schleswig Holstein (UKSH, Kiel, Germany). Mice were housed in groups of up to five littermates in individually ventilated cages (Green Line, Techniplast) under specific pathogen-free conditions. Mice were euthanized by cervical dislocation, and tissues were collected for histological and molecular analyses.

Conditional-ready *Hkdc1*^tm1a(KOMP)Wtsi^ mice were purchased from the KOMP repository ([https://www.komp.org](https://www.komp.org/), clone ID 799725, C57BL/6N-A^tm1Brd^ background). These mice were generated using a conditional-ready exon-trapping strategy. In brief, a neomycin selection cassette was inserted into the *Hkdc1* genomic locus between exons 2 and 5. The cassette contained an FRT site followed by a lacZ sequence, a loxP-flanked neomycin resistance gene with an internal FRT site, and a third loxP site downstream of targeted exons 3-4 (Supplementary Figure S3). These *tm1a* offspring were bred with a flp-deleter strain purchased from Jackson Laboratory ([https://www.jax.org](https://www.jax.org/), Stock Number: 011065) to remove the neomycin selection cassette, generating the final conditional *tm1c* allele (Supplementary Figure S3). To generate the *Hkdc1*^∆IEC^ mouse line (alternatively termed *Hkdc1*^fl/fl^-*Villin*::Cre^+^ or “HKDC1”), we crossed the conditional *tm1c* allele-carrying mice with a strain expressing CRE recombinase under the control of the IEC-specific *Villin* promoter (purchased from Jackson Laboratory, Stock Number: 021504). Littermate *Hkdc1*^fl/fl^ mice without CRE recombinase were used as WT controls. For *in vivo* analysis of HKDC1 function in intestinal carcinogenesis, *Apc*^Min/+^ (Adenomatous-polyposis-coli multiple intestinal neoplasia) mice were purchased from Jackson Laboratory (Stock Number: 002020) and crossed with *Hkdc1*^∆IEC^ mice to generate tumor-bearing *Apc*^Min/+^-*Hkdc1*^∆IEC^ mice. *Apc*^Min/+^ mice serve as a standard model for sporadic intestinal carcinogenesis [5]. *Apc*^Min/+^ mice carry a mutation in the *Apc* gene, which encodes a truncated, nonfunctional APC protein [6]. APC normally functions as a critical tumor suppressor by promoting β-Catenin degradation within the Wnt signaling pathway [7], thereby inhibiting the activation of tumorigenic target genes such as *c-myc*.

**Xenograft mouse model**

To generate subcutaneous xenografts, WT or HKDC1-deficient Caco-2 cells (2 x 10^6^ cells in 100 µl PBS) were injected into the right flank of 8- to 12-week old male NOD.Cg-Prkdc^SCID^ Il2rg^tm1Wjl^/SzJ (NSG) mice purchased from Charles River Germany [8]. Viability of the injected Caco-2 cells was confirmed before and after injection using plating and PI staining (Logos Biosystems, catalogue number F23001, Lot AP0BBH2901). Mice were checked for disease symptoms multiple times per week. Tumor growth and volume were traced assessed using caliper measurements. For ethical reasons, the experiment was discontinued on day 70 when tumors in the WT group reached the endpoint volume of 1500 mm^3^. Mice were euthanized by cervical dislocation, and the subcutanous tumors were dissected, measured to determine tumor volume, and weighed to assess tumor mass.

**Histology and immunostaining**

For immunohistochemistry, intestines were flushed with phosphate buffered saline (PBS), cut open longitudinally, and rolled from distal to proximal. Swiss rolls were fixed in 10% formalin solution (ThermoFisher Scientific) over night at 4 °C and then embedded in paraffin. 5 µm thick sections were cut and stained with hematoxylin and eosin (H&E) or subjected to immunostaining using the Vectastain ABC kit (Vector Laboratories), including antigen retrieval in boiling citrate buffer. Primary antibodies were incubated overnight. For immunostaining of Ki67, we used a 1:500 diluted mouse anti-Ki67 antibody (BD Biosciences, cat.no. 556003). The TUNEL assay was performed using the ApopTag Plus Peroxidase In Situ Apoptosis Detection Kit (Merck Millipore) according to the manufacturer’s instructions.

For immunofluorescence staining of mouse tissue, 5 µm thick sections of intestinal Swiss roles blocked with PBS containing 5 % BSA and 0.2 % TritonX. Primary antibodies (goat-anti-E-cadherin, 1:500, #3195, Cell Signaling Technology; rabbit-anti-HKDC1, 1:500, #ab228729, Abcam) were incubated over-night in PBS containing 1 % BSA. After washing, secondary antibodies were added (Alexa Fluor 488 goat anti mouse, Invitrogen, A32731 and Alexa Fluor 555 goat anti rabbit, Invitrogen, A21430) and DNA counterstaining was performed using DAPI (1:40,000 in PBS, D9542, Sigma Aldrich). Slides were then mounted using antifade mounting media (DAKO, Hovedstaden, Denmark).

For immunofluorescence staining of cultured cells, Caco-2 and CMT-93 cells were seeded on poly-l-lysin (#P4707, Sigma-Aldrich) coated cover slips in cell culture medium. After stimulation with STS, cells were washed with PBS and fixed in 4 % paraformaldehyde (pH 7.4, #158127, Merck), followed by blocking in PBS containing 2 % BSA. Primary anti-ß-actin (1:1000 in 0.1 % BSA, A-5441, Sigma-Aldrich) and either anti-HK1 (1:500 in 0.1 % BSA, #HPA011956, Sigma-Aldrich), anti-HK2 (1:500 in 0.1 % BSA, #NBP16814, Novus Biological), or anti-HKDC1 (1:500 in 0.1% BSA, #ab228729, Abcam) antibodies were incubated overnight. Secondary antibody incubation, DNA counterstaining and mounting was performed as described for Swiss roles.

Slides were visualized using a Zeiss Imager Z1 microscope (Zeiss) and pictures were taken using ZEN pro software (Zeiss, version 3.4). Proliferative and apoptotic cells were quantified by counting Ki67- and TUNEL-positive cells in at least 30 randomly selected crypts per sample in a blinded fashion, with the mouse identity masked during the counting process. Fluorescence signal intensity was quantified from images using Fiji/ImageJ software.

**Isolation and culture of intestinal organoids**

Organoids were generated from intestinal crypts of *Hkdc1*^∆IEC^ and tumorous tissue of *Apc*^Min/+^-*Hkdc1*^∆IEC^ mice following established procedures as described before [9]. The desired number of cells was plated in a 50 µl drop of a 1:1 mixture of Advanced DMEM/F12 and Matrigel (BD). ENR (epidermal growth factor [EGF], Noggin, and R-Spondin)-conditioned medium consisted of 70 % (*v*/*v*) 2 × basal medium (Advanced DMEM/F12 supplemented with HEPES [4-(2-hydroxyethyl)-1-piperazineethanesulfonic acid, 1 mol/L], Glutamax [100×], Penicillin/streptomycin 10,000 U/mL [1:50] and N-Acetylcysteine [500 mmol/L]), 10 % (*v*/*v*) Noggin-conditioned medium and 20 % R-Spondin-conditioned medium. Noggin- and R-Spondin-conditioned media were prepared as described below. Organoids were cultivated in 24-well plates at 37°C with 5 % CO_2_ atmosphere in Matrigel (BD) with ENR-conditioned medium supplemented with 0.1 % human recombinant EGF (50 µg/mL). The medium was changed every two to three days, and organoids were passaged every four to eight days. Organoids were cultured for at least two passages before being used for experiments.

**Organoid** **forming** **assay**

Organoids were resuspended in 1 ml TrypLE Express (Invitrogen) supplemented with 10 µmol/L Y-27632 (ThermoFisher Scientific) and incubated at 37 °C to create single cells. The desired cell number (5000 cells per 20 µl and well) was cultured in a 24-well plate. After a 5-day growth period, organoids were counted, and diameters were measured using ZEN software (version 3.4).

**Cell culture**

Caco-2 and CMT-93 cells were purchased from DSMZ (ACC-169) and ATCC (CCL-223), respectively. Cells were cultured at 37 °C and 5 % CO_2_ in their respective media (Caco-2: MEM + 20 % Fetal bovine serum [FBS]; CMT-93: DMEM + 10 % FBS) until a fully confluent cellular monolayer was established. Cells were stimulated at 70 % confluency.

HEK Noggin cells [10], kindly provided by Prof. Zeissig (CRTD, Dresden University, Germany), were cultured for three cycles in DMEM containing 10 % FBS and 10 µg/ml Puromycin (Sigma-Aldrich). Afterwards, cells were diluted 1:20 in DMEM + 10 % FBS and cultured for four days. The supernatant was then collected and centrifuged to eliminate cells. This process was repeated after four additional days of culturing, and both batches were combined. HEK R-Spondin-1-producing cells [10], kindly provided by Prof. Zeissig (CRTD, Dresden University, Germany), were cultured for three cycles in DMEM containing 10 % FBS and 300 g/ml Zeocin (Thermo Fischer Scientific). Following a 1:20 dilution in DMEM + 10 % FBS, the cells were cultured for four days. The media was collected and centrifuged to remove cells. This process was repeated after four additional days of culturing, and both batches were combined.

**Generation and culture of HKDC1-deficient Caco-2 and CMT-93 cells**

HKDC1-deficient cell lines were generated using the CRISPR/Cas9 technique with plasmids assembled using the GeneArt™ CRISPR Nuclease Vector with CD4 Enrichment Kit (Thermo Fisher Scientific), following the manufacturer’s instructions with the following primers: CRISPR_Caco-2_For (5’-TTC CCG CGG ATG ATT TCA TTG TTT T-3’), CRISPR_Caco-2_Rev (5’-AAT GAA ATC ATC CGC GGG AAC GGT G-3’), CRISPR_CMT-93_For (5’-CCT GTA TCA CAT GCG GCT CTG TTT T-3’), and CRISPR_CMT-93_Rev (5’-AGA GCC GCA TGT GAT ACA GGC GGT G-3’). Correct plasmid sequences were validated using in-house Sanger sequencing. Caco-2 or CMT-93 cells were transfected with the respective *HKDC1* or *Hkdc1* CRISPR plasmid using the Lipofectamine 3000 reagent kit (Thermo Fisher Scientific). Positive clones were purified using the Dynabeads® CD4 Positive Isolation Kit (Thermo Fisher Scientific), followed by thorough washing until only directly beads-bound cells remained, with no secondary bindings, as monitored by stereomicroscopic evaluation. Beads were detached, cell numbers were counted, and single cells were seeded into individual wells of a 96-well plate. Single colonies were picked, expanded, and screened for HKDC1 deficiency via western blot analysis, generating monoclonal HKDC1-deficient Caco-2 and CMT-93 cell clones. Caco-2 or CMT-93 cells subjected to the CRISPR transfection and selection procedure but still expressing HKDC1 (as confirmed by western blot) were used as WT controls.

***In vitro* cell formation assays**

The proliferation of WT and HKDC1-deficient Caco-2 and CMT-93 cells was assessed using multiple methodologies: estimating cell density or measuring protein content as a proxy for cell count. To estimate cell density, Caco-2 or CMT-93 cells from regular culture were detached and initially counted using the Cellometers Auto T4 Plus (Heraeus). A fixed number of cells (CMT-93: 1 x 10^6^, Caco-2: 1.5 x 10^6^) was seeded in 24-well plates, and four days after seeding, the cell count was determined again to monitor growth. Alternatively, protein was extracted as described below and used as a molecular measure of cell count. Proteins were extracted from cell pellets or scrapings of small intestinal mucosa from *Hkdc1*^∆IEC^ mice and WT littermate controls. Samples were lysed in ice-cold RIPA buffer containing protease and phosphatase inhibitors and were homogenized by sonication (for cells) or bead beating (for tissue) using a tissue lyser (Qiagen). After centrifugation, the supernatant was used for protein concentration measurement. The entire procedure was conducted at 4 °C or on ice. Protein was then quantified using the BCA assay (BioRad), according to the manufacturer’s instructions on a M200 Pro microplate reader (Tecan).

**Cell death quantification**

Cell death was assessed using viability staining with zombie red (BioLegend), which selectively stains dead cells due to their compromised cell membranes, followed by FACS analysis. Caco-2 and CMT-93 cells or organoids were cultured as described above in 24-well plates. Cells were stimulated with staurosporine (10 µmol/L for Caco-2, 2 µmol/L for CMT-93 cells and organoids), TNF (100 ng/ml), or IFN-β (50 ng/ml) for 24 hours to induce cell death. Stimulated cells and organoids were washed and dissociated into single cells as described. The cells were then resuspended in 100 µl of FACS wash buffer (1 % FBS in PBS), transferred to a 96-well plate, and stained with zombie red (BioLegend, 1:1000 dilution in FACS wash buffer) for 30 minutes at room temperature in the dark. After washing, cells were resuspended in 100 µl of FACS wash buffer and subjected to FACS analysis using the Spectral Cell Analyzer (SONY). The percentage of dead cells was determined based on Zombie red intensity, after excluding doublets and debris using the Sony SA3800 software (version 2.0.5).

**Mitochondrial membrane potential**

Mitochondrial membrane potential was assessed using the MitoProbe tetramethylrhodamine-methyl ester (TMRM) Assay-Kit (Thermo Fisher Scientific). TMRM is a positively charged dye that translocate across the mitochondrial membrane and accumulates in the negatively charged mitochondrial matrix [11]; reduced TMRM staining indicates a disrupted mitochondrial membrane potential. Caco-2 and CMT-93 cells were cultured overnight, and organoids were harvested on the day of staining. TMRM was added at a final concentration of 20 nmol/L and incubated for 30 minutes at 37 °C in a 5 % CO_2_ atmosphere. After a brief wash, Caco-2 and CMT-93 cells were trypsinzed, whereas organoids were incubated with TrypLE Express (Thermo Fisher Scientific) for 15 minutes to generate single cells. The single cell solutions were centrifuged at 300 x g for 5 minutes, washed, and resuspended in FACS wash buffer. All cells were analyzed using the Spectral Cell Analyzer (SONY). The percentage of TMRM-positive cells was determined after excluding doublets and debris in the Sony SA3800 software (version 2.0.5).

**Seahorse metabolic assays**

The mitochondrial activity of Caco-2 and CMT-93 cells, as well as intestinal organoids, was measured in real-time using the Seahorse XF Cell Mito Stress Test Kit according to the manufacturer’s instructions on a Seahorse XFe24 Analyzer (Agilent Technologies). Three independent experiments were performed with *n* = 9 replicates. For Caco-2 or CMT-93, 4 x 10^4^ cells were seeded 24 hours before the assay. For intestinal organoids, 5000 cells were seeded as a 20 µl drop in pure Matrigel onto the Agilent Seahorse XF24 cell culture microplate before 400 µl of organoid culture medium was added, and the assay was performed as previously described [12].

**RNA isolation and qPCR**

Total RNA was extracted from cell pellets, organoids, or intestinal tissue using the RNeasy Mini Kit (Qiagen) according to the manufacturer’s protocol. RNA concentration was measured using a NanoDrop ND-1000 spectrophotometer (PeqLab Biotechnologie). Total RNA was reverse-transcribed into cDNA using the Maxima H Minus First Strand cDNA Synthesis Kit (ThermoFisher Scientific). qPCR was carried out using SYBR Select Master Mix (Applied Biosystems) according to the manufacturer’s instructions on a Viia 7 Real-Time PCR System (ThermoFisher Scientific). Expression levels were normalized to *Actb* (β-actin). Primer sequences for qPCR are listed in Supplementary Table S2.

**Transcriptional profiling by RNA sequencing**

Total RNA from intestinal organoids of *Apc*^Min/+^-*Hkdc1*^∆IEC^ and WT littermate mice were extracted using the RNeasy Mini Kit (Qiagen) according to the manufacturer’s protocol. RNA concentration and integrity were analysed using a TapeStation 4200 System (Agilent) and a Qubit 4 fluorometer (ThermoFisher Scientific). RNA-sequencing libraries were prepared using the TruSeq® RNA seq Library Prep Kit v2 according to the Illumina TruSeq® messenger (mRNA) sequencing protocol. The RNA-seq libraries were sequenced on an Illumina NovaSeq 6000 sequencer (Illumina, San Diego,CA) with an average of 15 million paired-end reads (2 x 150 bp) at the IKMB NGS core facilities. Adapter sequences were removed from the raw reads using cutadapt (v2.8) with a minimum overlap of 3 bp and a maximum error tolerance of 10% mismatches (TrueSeq forward adapter and TruSeq reverse complement universal adapter). Additional 3’-end quality trimming was performed to a minimum Phred score of 25, and poly-G ends were trimmed to address dark-cycle issues associated with Illumina’s two-color chemistry, utilizing the cutadapt option --nextseq-trim=25. An additional quality filtering step was applied using PrinSeq Lite (v0.20.4) to achieve a mean read quality of at least Phred score 30 across all bases, with a maximum of 5 unknown base calls and a minimum read length of 30 bp. Post-QC read qualities were visually inspected using FastQC (v0.11.7). The filtered reads were then mapped to the Mus musculus reference genome GRCm38, released by the European Bioinformatics Institute (version 99, February 2020), using Hisat2 (v2.1.0) software. Only uniquely mapped reads were retained utilizing SamTools (v1.9) with the -F 256 flag. Gene and exon abundances were then counted for properly mapped read pairs, with strandedness information (-s 2) taken into account, using the “featureCounts” tool from the subread software (v2.0.1). Samples that did not meet the QC standards were excluded from further analysis. Count data were analysed in R (version 4.4.0 [13]) using the DESEq2 R package (version 1.44.0 [14]) to obtained differentially expressed (DE) genes. Genes with less than 10 counts across all samples were removed before analysis. After comparing KO vs. WT samples within the respective tissues, genes with an adjusted *p*-value (FDR) below 0.05 were retained for further analysis. Subsequently, Gene Ontology Biological Process (BP) enrichment was performed in R with the ClusterProfiler package (version 4.12.0 [15]) using annotations in the org.Mm.eg.db package (version 3.19.1 [16]). Genes with positive and negative log2 fold changes were enriched separately for each comparison.

**HKDC1 immunoprecipitation and liquid chromatography-mass spectrometry**

Protein lysates from small intestinal mucosal scrapings of HKDC1^∆IEC^ and WT littermate mice were used for Immunoprecipitation (IP). 1 µg of anti-HKDC1 antibody (Abcam, ab2279278) was bound to 50 µL Dynabeads (Thermo Fisher Scientific, 10004D) by incubation at 4 °C for 1 hour and then crosslinked in 250 µl 5 mol/L bis(sulfosuccinimidyl)suberate for 30 minutes at room temperature. The crosslinking reaction was stopped by adding 12.5 µl quenching buffer (1 mol/L Tris-HCl, pH 7.5) and incubating at room temperature for 15 minutes. After washing, 200 µl protein lysate was added to the crosslinked beads and incubated at 4 °C overnight. The beads were washed and transferred into a fresh tube. Elution was performed by adding 22 µl elution buffer (50 mmol/L Glycine, pH 2.8) and incubating for 5 minutes at room temperature. 10 µl of Tris (pH 7.5) was added, and samples were stored at -20 °C. The IP eluates were analyzed using liquid chromatography-mass spectrometry (LC-MS). Briefly, sample cleanup was performed according to the SP3 protocol [17]. The samples were first reduced with 10 mmol/L dithiothreitol at 56 °C for 1 hour, followed by alkylation with 50 mmol/L iodoacetamide in the dark at 20 °C for 30 minutes. Proteins were then precipitated onto hydrophilic and hydrophobic Sera-Mag SpeedBeads (carboxylate-modified magnetic beads, hydrophilic: 45152105050250, hydrophobic: 65152105050250, GE Life Sciences) using a 6-fold volume of ethanol. After three washes with 80 % ethanol, the samples were digested with trypsin (enzyme:protein ratio of approximately 1:40) overnight at 37 °C. Peptides were acidified to pH 2-3 using trifluroacetic acid (TFA) prior to LC-MS analysis on a Dionex U3000 UHPLC system (ThermoFisher Scientific) equipped with a column chromatography setup coupled to a Q Exactive Plus Orbitrap MS (ThermoFisher Scientific), using a nanospray ion source with a 1.7 kV spray voltage and a 250°C capillary temperature with a 20 µm Tip emitter (MS Wil). Peptides were concentrated and washed onto a trap column (75 μm × 2 cm, 2 μm C_18_ resin, 100 Å; Acclaim PepMap100, Thermo Fisher Scientific) for 5 minutes with 2% ACN and 0.05% aqueous TFA at a flow rate of 30 µl/minute. Subsequently, peptides were separated on an analytical column (75 µm x 50 cm, 2 μm C_18_ resin, 100 Å; Acclaim PepMap100, Thermo Fisher Scientific) at 300 nL/minute using a 60 minutes linearly increasing gradient of LC solvent B (80 % acetonitril (ACN), 0.1 % formic acid (FA)) in LC solvent A (0.1 % FA). The linear gradient was followed by a sharp increase to 90 % solvent B for 5 minutes, an isocratic 10 minutes washing step, and finally a column equilibration with 5 % B for 12 minutes. After a delay time of 5 minutes, the MS acquisition program consisted of a full-scan MS (range: 300 – 1,800 m/z, resolution: 60,000, automatic gain control [ACG] target: 3e6, maximal injection time [IT]: 50 ms) with the top 10 MS/MS acquisition of the most intense ions using a 2.0 m/z isolation (resolution: 15,000, AGC target: 1e5, maximal IT: 50 ms). Ions of unassigned, +1, and > +8 charge states were excluded. For fragmentation, higher-energy collisional dissociation (HCD) was utilized with a normalized collision energy (NCE) of 27. Dynamic exclusion (20 s) and lock mass (445.12003 m/z) were enabled. Each sample was analyzed using two technical replicates. Raw MS data were searched against the UniProt reference proteome of *Mus musculus* strain C57BL/6J (55.315 entries, 28. March.2022) and common contaminants (cRAP, contact/dust and laboratory contaminants; 42 entries) using Proteome Discoverer (version 2.2.0.388; ThermoFisher Scientific). Percolator was used for posterior error calculation [18], combining the results of the database searches restricted by *q*-value to FDR ≤ 0.01. A given protein was considered as “identified” when a valid MS^2^ spectrum was available for at least one of the peptides belonging to that protein. Detailed settings are listed in Supplementary Tables S3 and S4. Structural proteins such as cytoskeletal proteins (myosin, actin, tubulin, tropomodulin), keratins, histones, and ribosomal proteins, along with immunoglobulin proteins and proteins with a coverage of less than 25%, were removed from further analyses. To generate a protein-protein interaction network and visualize functional hubs among the identified HKDC1 interaction partners, the Search Tool for the Retrieval of Interacting Genes (STRING: <https://string-db.org/> (version 12.0, accessed on 22 February 2024)) was used [19] in medium confidence (0.400) mode.

**Statistics**

General statistical analyses were performed using GraphPad Prism 9 (GraphPad Software Inc., La Jolla, USA). For pairwise comparisons, the Mann-Whitney-U-test was used, whereas for multiple comparisons, one-way or two-way ANOVA with false discovery rate (FDR) correction were performed. Data are shown as mean ± standard error of the mean (SEM). A *P*-value < 0.05 was considered significant (*). A *P*-value < 0.01 was considered strongly significant (**), and *P*-values of < 0.001 (***) and < 0.0001 (****) were considered highly significant.

**Supplementary References**

1. Uhlén M, Fagerberg L, Hallström BM, Lindskog C, Oksvold P, Mardinoglu A, et al. Tissue-based map of the human proteome. Science. 2015;347:1260419.

2. Hutter C, Zenklusen JC. The Cancer Genome Atlas: creating lasting value beyond its data. Cell. 2018;173:283–5.

3. The Cancer Genome Atlas Research Network, Weinstein JN, Collisson EA, Mills GB, Shaw KRM, Ozenberger BA, et al. The Cancer Genome Atlas pan-cancer analysis project. Nat Genet. 2013;45:1113–20.

4. Lonsdale J, Thomas J, Salvatore M, Phillips R, Lo E, Shad S, et al. The Genotype-Tissue Expression (GTEx) project. Nat Genet. 2013;45:580–5.

5. Uronis JM, Threadgill DW. Murine models of colorectal cancer. Mamm Genome. 2009;20:261–8.

6. Moser AR, Luongo C, Gould KA, McNeley MK, Shoemaker AR, Dove WF. ApcMin: a mouse model for intestinal and mammary tumorigenesis. Eur J Cancer. 1995;31A:1061–4.

7. Schneikert J, Behrens J. The canonical Wnt signalling pathway and its APC partner in colon cancer development. Gut. 2007;56:417–25.

8. Czulkies BA, Mastroianni J, Lutz L, Lang S, Schwan C, Schmidt G, et al. Loss of LSR affects epithelial barrier integrity and tumor xenograft growth of CaCo-2 cells. Oncotarget. 2017;8:37009–22.

9. Sato T, Stange DE, Ferrante M, Vries RGJ, Van Es JH, Van Den Brink S, et al. Long-term expansion of epithelial organoids from human colon, adenoma, adenocarcinoma, and barrett’s epithelium. Gastroenterology. 2011;141:1762–72.

10. Miyoshi H, Stappenbeck TS. In vitro expansion and genetic modification of gastrointestinal stem cells in spheroid culture. Nat Protoc. 2013;8:2471–82.

11. Creed S, McKenzie M. Measurement of mitochondrial membrane potential with the fluorescent dye tetramethylrhodamine methyl ester (TMRM). Methods Mol Biol. 2019;1928:69-76

12. Ludikhuize MC, Meerlo M, Burgering BMT, Rodríguez Colman MJ. Protocol to profile the bioenergetics of organoids using Seahorse. STAR Protocols. 2021;2:100386.

13. R Core Team. R: A language and environment for statistical computing. Vienna, Austria: R Foundation for Statistical Computing; 2024.

14. Love MI, Huber W, Anders S. Moderated estimation of fold change and dispersion for RNA-seq data with DESeq2. Genome Biol. 2014;15:550.

15. Wu T, Hu E, Xu S, Chen M, Guo P, Dai Z, et al. clusterProfiler 4.0: A universal enrichment tool for interpreting omics data. The Innovation. 2021;2:100141.

16. Carlson M. org.Mm.eg.db: Genome wide annotation for mouse [Internet]. 2024. Available from: https://doi.org/doi:10.18129/B9.bioc.org.Mm.eg.db

17. Hughes CS, Moggridge S, Müller T, Sorensen PH, Morin GB, Krijgsveld J. Single-pot, solid-phase-enhanced sample preparation for proteomics experiments. Nat Protoc. 2019;14:68–85.

18. Käll L, Storey JD, MacCoss MJ, Noble WS. Assigning Significance to Peptides Identified by Tandem Mass Spectrometry Using Decoy Databases. J Proteome Res. 2008;7:29–34.

19. Szklarczyk D, Kirsch R, Koutrouli M, Nastou K, Mehryary F, Hachilif R, et al. The STRING database in 2023: protein–protein association networks and functional enrichment analyses for any sequenced genome of interest. Nucleic Acids Res. 2023;51:D638–46.

**Supplementary Figure Legends**


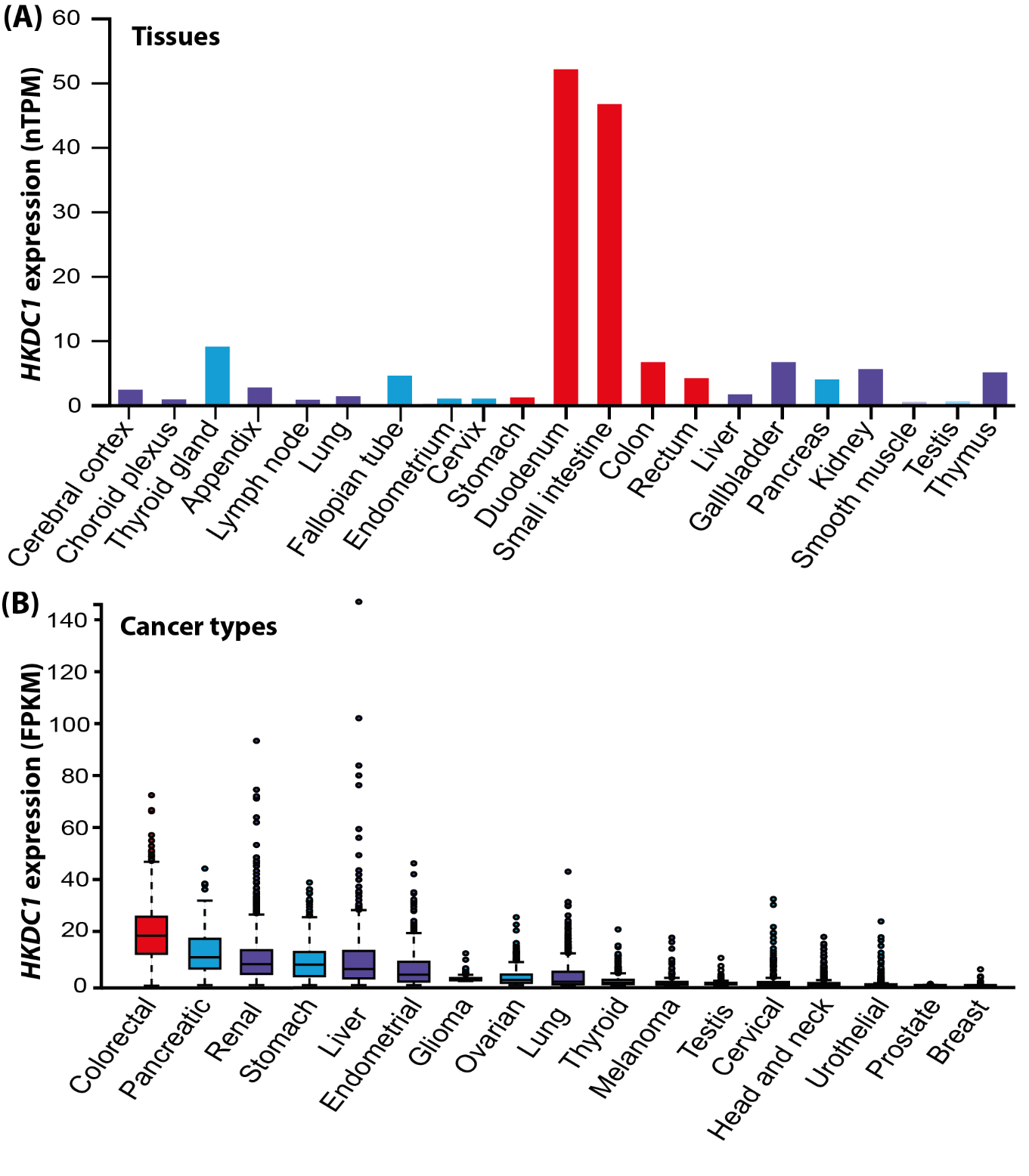


**Supplementary Figure S1. *HKDC1* expression is associated with colorectal cancer.**

(A) *HKDC1* expression in nTPM (normalized protein-coding transcripts per million) across different organs based on publicly available RNA-seq data from the Human Protein Atlas (HPA).

(B) *HKDC1* expression in FPKM (Fragments Per Kilobase per Million reads) from RNA-seq data across 17 cancer types from The Cancer Genome Atlas (TCGA, https://[www.cancer.gov/ccg/research/genome-](http://www.cancer.gov/ccg/research/genome-)sequencing/tcga). The box plot depicts median values with the 0.25 and 0.75 percentiles. Outliers are defined as values exceeding 1.5 times the interquartile range.

Abbreviations: FKPM, Median Fragments Per Kilobase of exon per Million reads; HPA, Human Protein Atlas; , HKDC1, Hexokinase domain containing 1; nTPM, normalized protein-coding transcripts per million; RNA-seq, Ribonucleotide acid sequencing; TCGA, The Cancer Genome Atlas.


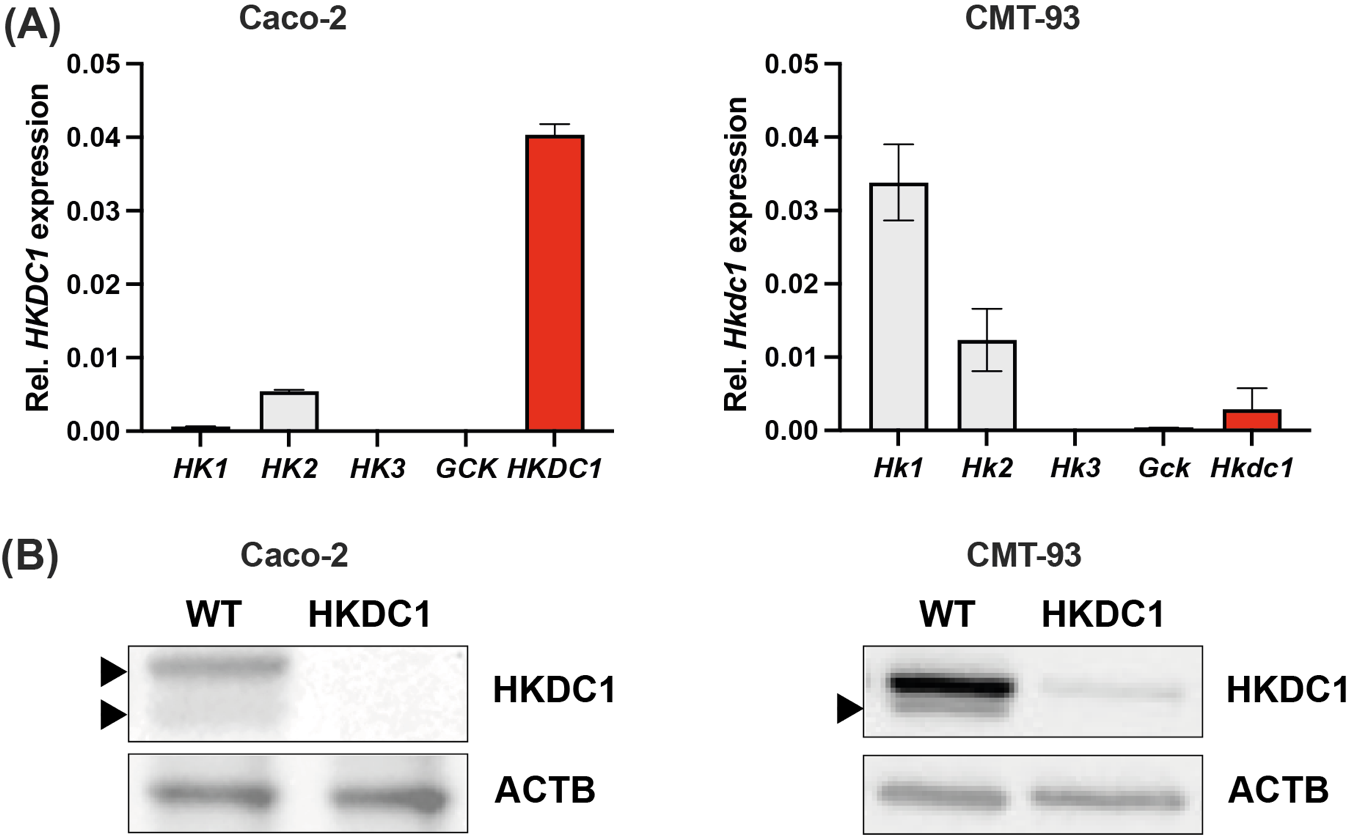


**Supplementary Figure S2. Expression of all hexokinases in the cell lines used.**

(A) Relative gene expression of all HK family members in human Caco-2 cells and murine CMT-93 cells, normalized to beta-Actin (*ACTB/Actb*) expression. GCK = Glucokinase*. n*= 5 per group.

(B) Western blot demonstrating effective HKDC1 deletion in human Caco-2 cells and murine CMT-93 cells. Arrows indicate the position of HKDC1 band.

Abbreviations: ACTB, beta Actin; GCK, Glucokinase; HK, Hexokinase; HKDC1, Hexokinase domain containing 1; WT, Wildtype.


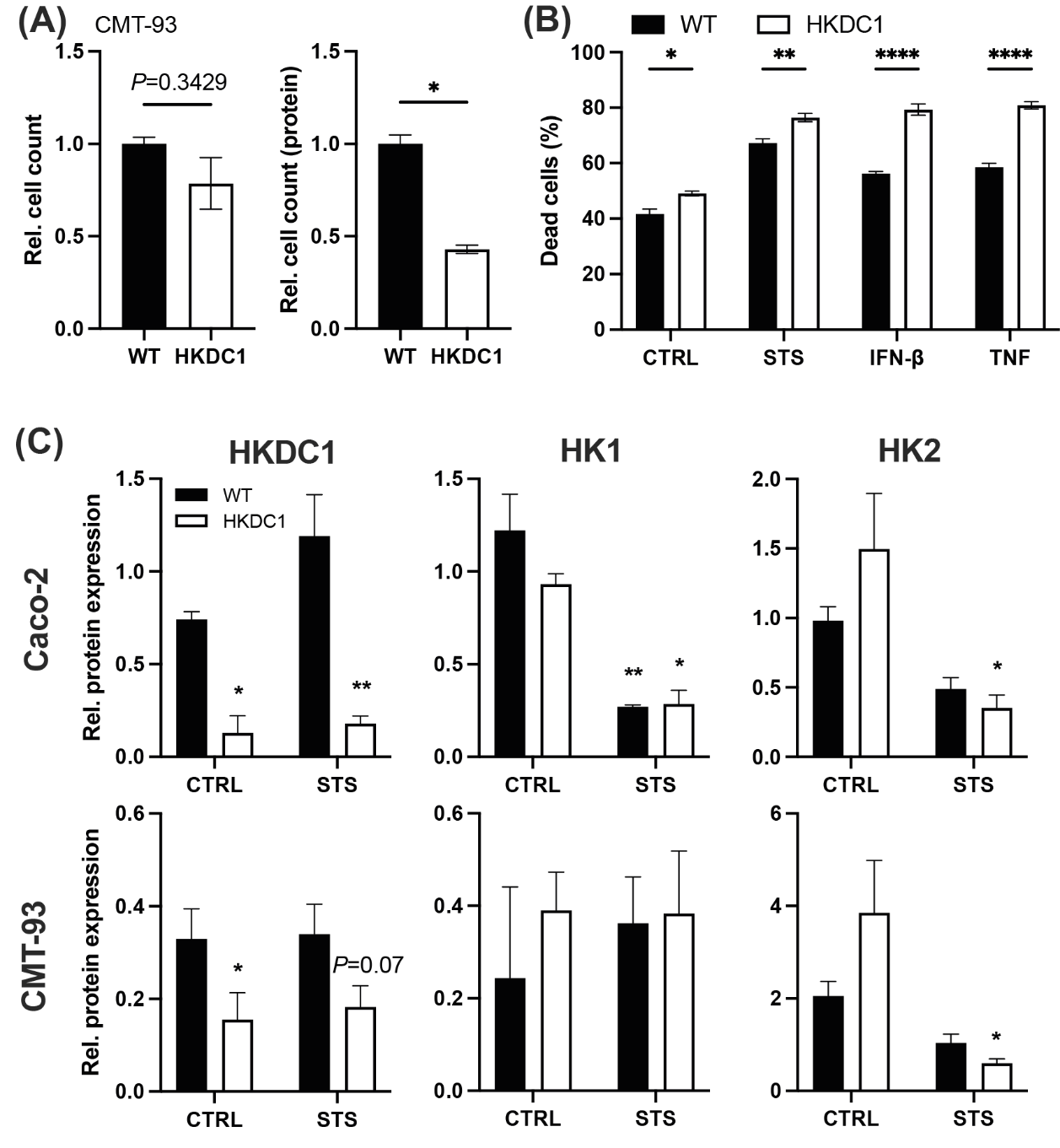


**Supplementary Figure S3. HKDC1 deletion alters proliferation and cell death.**

(A) Cell proliferation, measured by two independent methods (cell count and protein quantification), was reduced in HKDC1-deficient murine CMT-93 cells*. n*= 4 - 6 per group. **P*< 0.05, Mann-Whitney-U-test.

(B) WT and HKDC1-deficient CMT-93 cells were stimulated with either staurosporine (STS, 10 µmol/L for Caco-2 and 2 µmol/L for CMT-93 cells), tumor necrosis factor (TNF, 500 ng/µl), or interferon beta (IFN-β, 1000 U/µl) for 24 hours. Cell death was determined through zombie staining and FACS analysis. *n* = 5 per group. **P*< 0.05, ***P*< 0.01, and ****P*< 0.001, one-way ANOVA.

(C) HKDC1, HK1, and HK2 protein expression in WT and HKDC1-deficient Caco-2 and CMT-93 cells following STS treatment. No compensatory upregulation of HK1 or HK2 was detected in HKDC1-deficient cells. **P*< 0.05, and ***P*< 0.01 as per two-way ANOVA for WT vs. KO and CTRL vs. STS comparison. All data are presented as mean ± SEM.

Abbreviations: ANOVA, Analysis of variance; CTRL, Control; FACS, Fluorescent activated sell sorting; HK, Hexokinase; HKDC1, Hexokinase domain containing 1; IFN-β, Interferon beta; SEM, Standard error of the mean; STS, staurosporine; TNF, Tumor necrosis factor; WT, Wildtype.


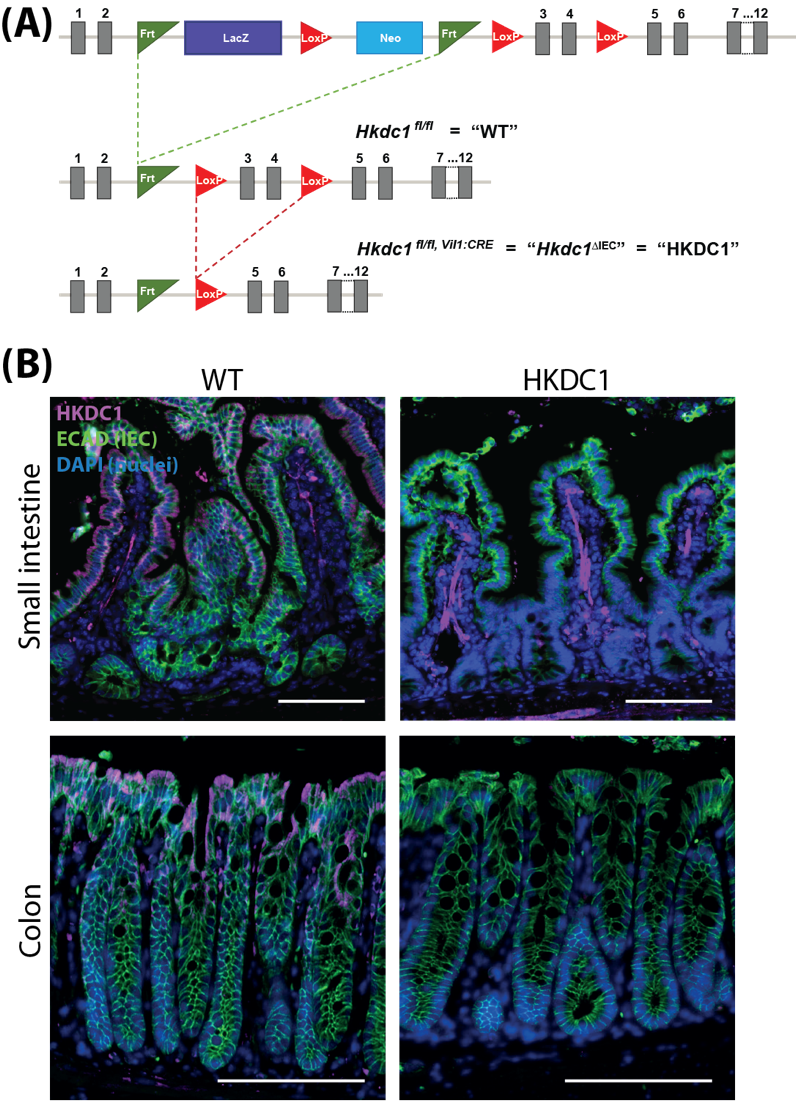


**Supplementary Figure S4. Generation of *Hkdc1*^∆IEC^ mice.**

(A) Genetic strategy for generating *Hkdc1*^∆IEC^ mice. IEC-specific HKDC1-deficient mice were generated by crossing conditional-ready *Hkdc1*^tm1a^ with Flp-deleter mice to create *Hkdc1* floxed (*Hkdc1^fl^*^/fl^) mice. These were then bred with Villin::CRE transgenic mice, which express CRE recombinase under the control of the IEC-specific *Villin1* promoter, resulting in *Hkdc1*^∆IEC^ mice carrying a specific conditional deletion of *Hkdc1* exons 3-4 specifically in IECs, leading to a non-functional HKDC1 protein.

(B) Knockout validation using immunofluorescence analyses for HKDC1 (purple), E-Cadherin (ECAD, IEC marker, green) and DAPI (nuclei, blue) counterstaining. HKDC1 protein is predominantly expressed in apical epithelial cells. Scale bar: 100 µm.

Abbreviations: DAPI, 4′,6-diamidino-2-phenylindole; ECAD, E-Cadherin; HKDC1, Hexokinase domain containing 1; IEC, Intestinal epithelial cell; WT, Wildtype.


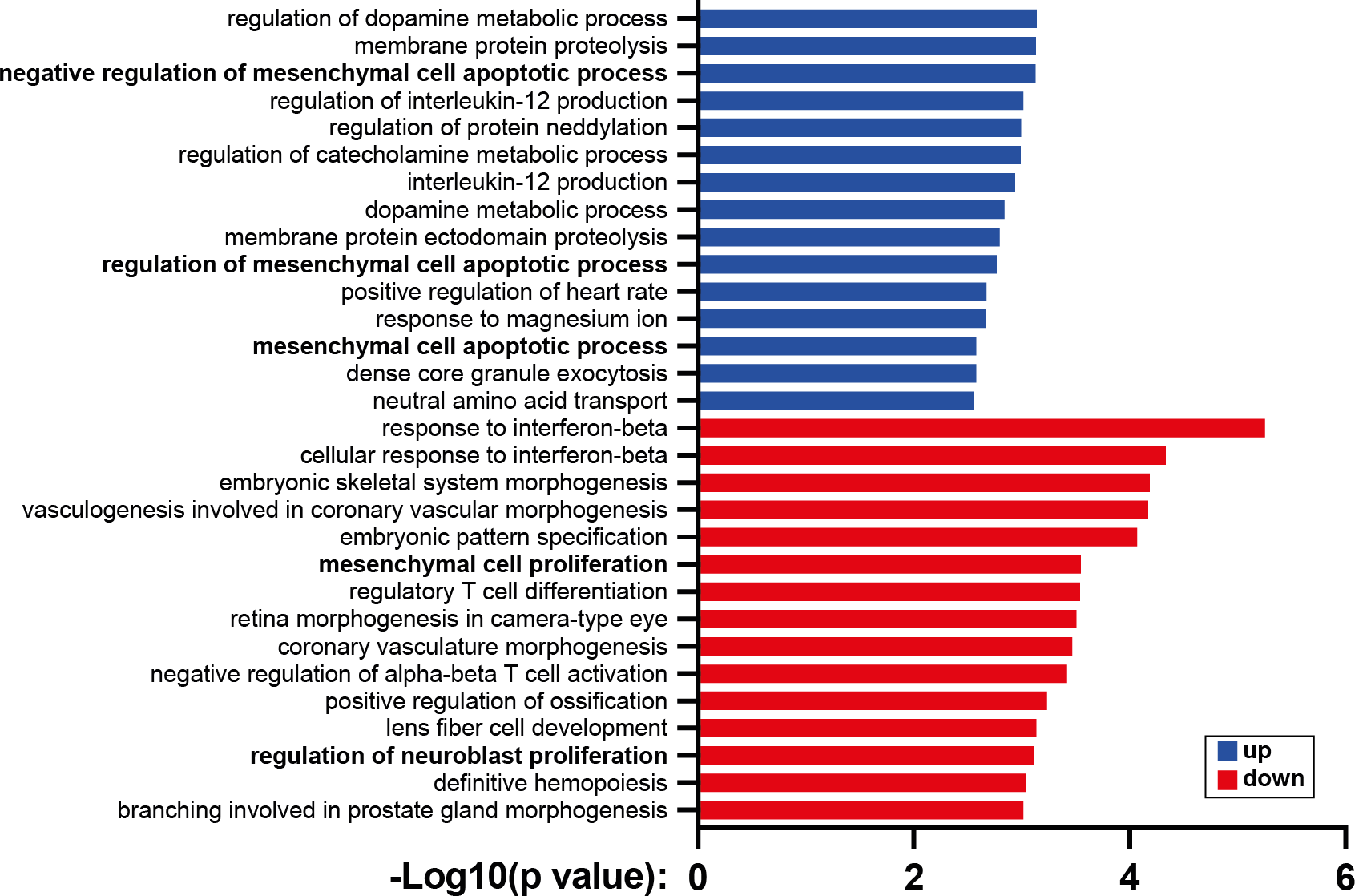


**Supplementary Figure S5. Transcriptional profiling reveals altered proliferation and cell death pathways in HKDC1-deficient intestinal organoids.**

Gene ontology (GO) biological process terms enriched among upregulated and downregulated genes in the transcriptomes of organoids derived from *Apc*^Min/+^-*Hkdc1*^∆IEC^ and littermate *Apc*^Min/+^-WT control mice. The 15 most enriched terms are shown, with proliferation- and apoptosis-related functions highlighted in bold.


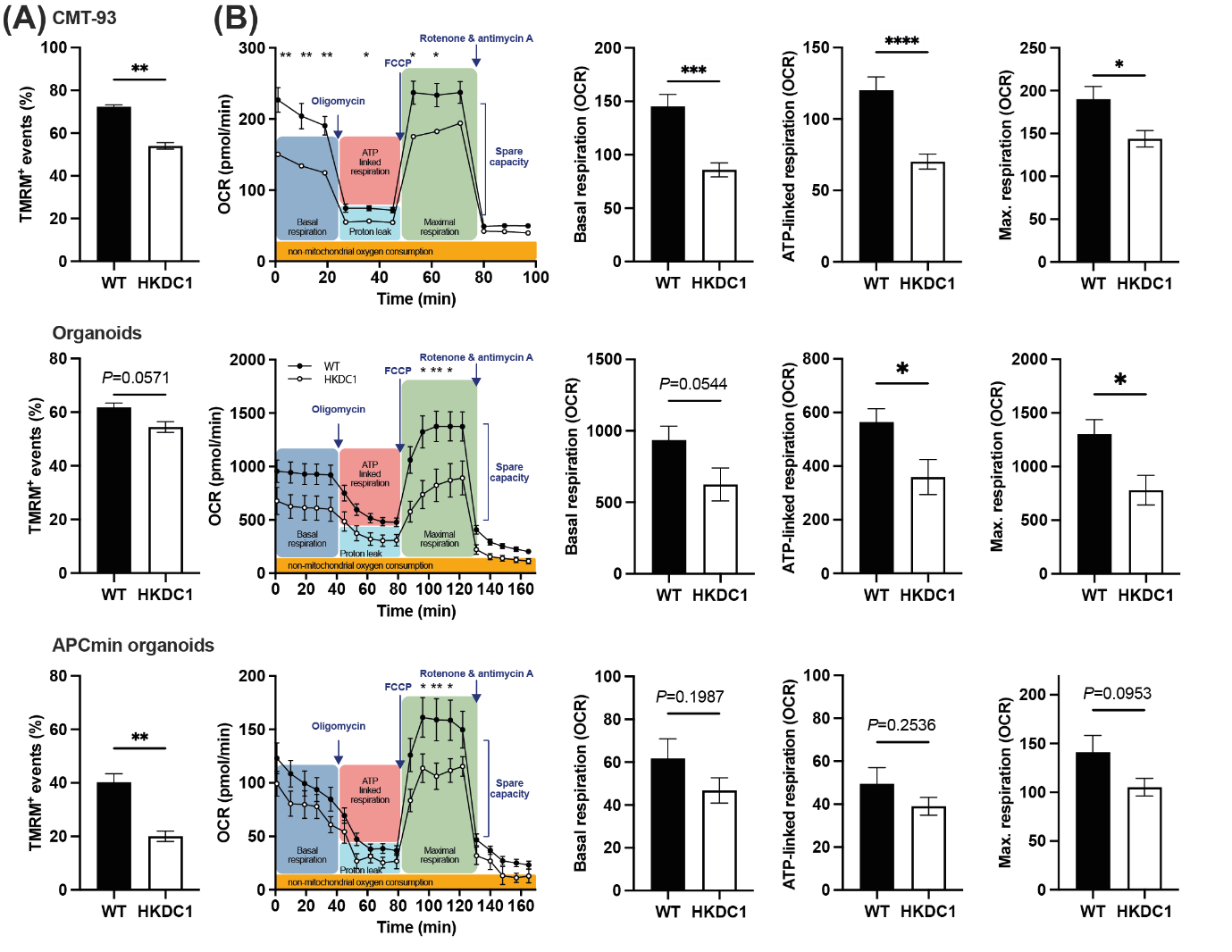


**Supplementary Figure S6. Lack of HKDC1 impairs mitochondrial function.** Mitochondrial phenotyping was performed in WT and HKDC1-deficient murine CMT-93 cells, as well as in normal and tumorigenic *Apc*^Min/+^ intestinal organoids.

(A) Deletion of HKDC1 reduced mitochondrial membrane potential, as assessed by TMRM staining and FACS analysis.

(B) oxygen consumption rate (OCR), a measure of mitochondrial activity, was reduced in *HKDC1*-deficient cells, as determined by Seahorse Mito Stress metabolic analysis. Basal respiration, maximal respiration, and ATP production were calculated from the depicted kinetic measurement*. n*= 9 - 10 per genotype and cell line/organoid. **P*< 0.05, ***P*< 0.01, ****P*< 0.001, and *****P*< 0.0001 as determined by the Mann-Whitney-U-test or two-way ANOVA. All data are shown as mean ± SEM.

Abbreviations: ANOVA, Analysis of variance; Apc^Min/+^, Adenomatous-polyposis-coli multiple intestinal neoplasia; ATP, Adenosine triphosphate; FACS, Fluorescence activated cell sorting; FCCP, carbonyl cyanide p-trifluoro methoxyphenylhydrazone; HKDC1, Hexokinase domain containing 1; OCR, Oxygen consumption rate; SEM, Standard error of the mean; TMRM, Tetramethylrhodamine-methyl ester; WT, Wildtype.


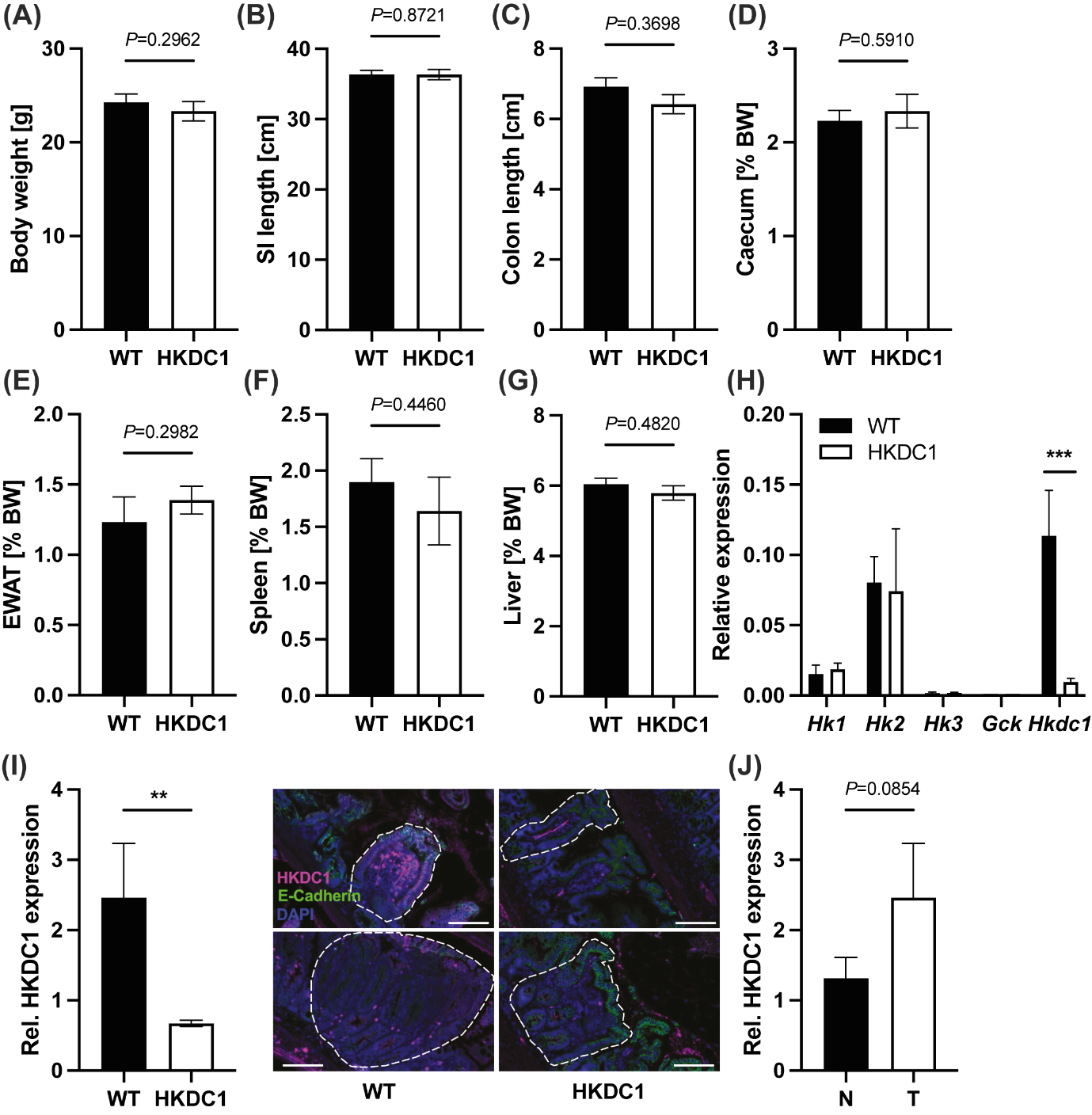


**Supplementary Figure S7. Organ measures did not differ between *Apc*^Min/+^-*Hkdc1*^∆IEC^** **mice and WT controls.**

(A-H) Organ measures were taken from 20-week-old *Apc*^Min/+^-*Hkdc1*^∆IEC^ mice and WT littermate controls.

(A) Body weight (BW).

(B) Small intestine (SI) length (cm).

(C) Colon length (cm).

(D) Caecum weight as a percentage of BW.

(E) Epididymal white adipose tissue (EWAT) as a percentage of BW.

(F) Spleen weight as a percentage of BW.

(G) Liver weight as a percentage of BW.

(H) Expression of all HK-family members in small intestinal tissue of *Apc*^Min/+^-*Hkdc1*^∆IEC^ mice and WT littermate controls. ****P*< 0.001. All data are shown as mean ± SEM.

(I-J) HKDC1 immunofluorescence in small intestinal tissue of *Apc*^Min/+^ mice (*n* = 5 per group). Data are shown as mean ± SEM.

(I) Reduced HKDC1 protein expression in tumors of *Apc*^Min/+^ mice lacking epithelial HKDC1. HKDC1 signal intensity was quantified and normalized to DAPI, with representative images shown. The dotted line indicates the measured tumor area. Scale bar: 100 µm. ***P* < 0.01 (Mann-Whitney-U-test).

(J) Elevated HKDC1 protein levels in tumor (T) compared to normal (N) tissue.

Abbreviations: Apc^Min/+^, Adenomatous-polyposis-coli multiple intestinal neoplasia; BW, Body weight; EWAT, Epididymal white adipose tissue; HK, Hexokinase; HKDC1, Hexokinase domain containing 1; IEC, Intestinal epithelial cell; OCR, Oxygen consumption rate; SEM, Standard error of the mean; SI, Small intestine; WT, Wildtype .

**Supplementary Table S1. HKDC1-interacting proteins identified by immunoprecipitation and included in the STRING interaction network.**

| **Protein name** | **Protein ID** | **Protein coverage** | **# peptides** | **# unique peptides** | **PSM** | **STRING protein identifier** |
| --- | --- | --- | --- | --- | --- | --- |
| ALDH1B1 | Q9CZS1 | 39 | 16 | 16 | 80 | 10090.ENSMUSP00000041260 |
| Arf1 | P84078 | 52 | 5 | 4 | 29 | 10090.ENSMUSP00000126120 |
| Arf4 | P61750 | 42 | 5 | 3 | 13 | 10090.ENSMUSP00000022429 |
| Arf5 | P84084 | 29 | 3 | 1 | 8 | 10090.ENSMUSP00000020717 |
| Atp5a1 | Q03265 | 56 | 24 | 24 | 223 | 10090.ENSMUSP00000026495 |
| Atp5b | P56480 | 56 | 16 | 16 | 124 | 10090.ENSMUSP00000026459 |
| C1ra | Q8CG16 | 28 | 15 | 15 | 44 | 10090.ENSMUSP00000063707 |
| C1s1 | E9Q6C2 | 30 | 14 | 7 | 39 | 10090.ENSMUSP00000125531 |
| Cfl1 | P18760 | 60 | 8 | 7 | 54 | 10090.ENSMUSP00000147514 |
| Clca1 | Q9D7Z6 | 37 | 26 | 26 | 129 | 10090.ENSMUSP00000029919 |
| Dbt | P53395 | 71 | 26 | 26 | 161 | 10090.ENSMUSP00000000349 |
| Des | P31001 | 76 | 37 | 32 | 363 | 10090.ENSMUSP00000027409 |
| Eif4a1 | P60843 | 39 | 10 | 10 | 48 | 10090.ENSMUSP00000127034 |
| Gapdh | A0A0A0MQF6 | 62 | 14 | 14 | 144 | 10090.ENSMUSP00000113942 |
| Gfpt1 | P47856 | 28 | 13 | 13 | 49 | 10090.ENSMUSP00000109288 |
| Hk1 | P17710 | 59 | 53 | 46 | 507 | 10090.ENSMUSP00000111946 |
| Hk2 | O08528 | 53 | 41 | 36 | 267 | 10090.ENSMUSP00000000642 |
| Hkdc1 | Q91W97 | 64 | 57 | 52 | 524 | 10090.ENSMUSP00000020277 |
| Hspa8 | P63017 | 39 | 19 | 15 | 128 | 10090.ENSMUSP00000015800 |
| Ldha | A0A1B0GSX0 | 26 | 9 | 9 | 71 | 10090.ENSMUSP00000148107 |
| Lgals2 | Q9CQW5 | 64 | 7 | 7 | 32 | 10090.ENSMUSP00000036598 |
| Lmna | P48678 | 59 | 31 | 30 | 140 | 10090.ENSMUSP00000029699 |
| Mptx2 | D3YYJ7 | 37 | 7 | 7 | 28 | 10090.ENSMUSP00000106855 |
| Phb | P67778 | 33 | 5 | 5 | 13 | 10090.ENSMUSP00000119603 |
| Prdx1 | P35700 | 42 | 6 | 4 | 36 | 10090.ENSMUSP00000114159 |
| Rack1 | P68040 | 32 | 7 | 7 | 41 | 10090.ENSMUSP00000020640 |
| Scin | Q60604 | 71 | 35 | 35 | 286 | 10090.ENSMUSP00000002640 |
| Slc25a4 | P48962 | 40 | 12 | 5 | 112 | 10090.ENSMUSP00000034049 |
| Slc25a5 | P51881 | 42 | 12 | 5 | 150 | 10090.ENSMUSP00000016463 |
| Vdac2 | Q60930 | 54 | 10 | 10 | 59 | 10090.ENSMUSP00000022293 |
| Vdac3 | Q60931 | 31 | 6 | 5 | 26 | 10090.ENSMUSP00000009036 |
| Vil1 | Q62468 | 80 | 55 | 55 | 1241 | 10090.ENSMUSP00000027366 |
| Vim | P20152 | 71 | 33 | 26 | 329 | 10090.ENSMUSP00000028062 |
| Zg16 | Q8K0C5 | 65 | 6 | 6 | 63 | 10090.ENSMUSP00000145876 |

**Proteins were quantified in the LFQ dataset, with structural proteins removed. PSM = peptide spectral match.**

**Supplementary Table S2. Sequences of forward (F) and reverse (R) primers used for RT-PCR analysis.**

| **Gene** | **Species** | **Sequence (5’-3’)** |
| --- | --- | --- |
| *Hk1* | Mouse | F: CGGAATGGGGAGCCTTTGG  R: GCCTTCCTTATCCGTTTCAATGG |
| *Hk2* | Mouse | F: CCCTGTGAAGATGTTGCCCACT  R: CCTTCGCTTGCCATTACGCACG |
| *Hk3* | Mouse | F: TGCTGCCCACATACGTGAG  R: GCCTGTCAGTGTTACCCACAA |
| *Gck (Hk4)* | Mouse | F: GAGATGGATGTGGTGGCAAT  R: ACCAGCTCCACATTCTGCAT |
| *Hkdc1* | Mouse | F: ATGTTTGCAGTACACTTGGTGG  R: AGGGTCTCATCCGAGAGCC |
| *Actb* | Mouse | F: GGCTGTATTCCCCTCCATCG  R: CCAGTTGGTAACAATGCCATGT |
| *HK1* | Human | F: CTGCTGGTGAAAATCCGTAGTGG  R: GTCCAAGAAGTCAGAGATGCAGG |
| *HK2* | Human | F: AAGGCTTCAAGGCATCTG  R: CCACAGGTCATCATAGTTCC |
| *HK3* | Human | F: GTGAGGTTGGGCTAGTTGTAGA  R: GTCCAGGGTATGGTCGAAGGT |
| *GCK (HK4)* | Human | F: GAATGACACGGTGGCCACGATG  R: CACTCGGTATTGACGCACATGCG |
| *HKDC1* | Human | F: GGCTTCACATTCTCATTTCC  R: TGTTGCTGCCTGTTCCTG |
| *ACTB* | Human | F: CATGTACGTTGCTATCCAGGC  R: CTCCTTAATGTCACGCACGAT |

**Supplementary Table S3. Proteome Discoverer settings-parameters.**

| **Spectrum Selector** | **General Settings** | **Precursor Selection** | **Use MS1 Precursor** |
| --- | --- | --- | --- |
| Spectrum Selector  Sequest HT | General Settings  Spectrum Properties Filter | Use new Precursor Reevaluation | True |
|  |  | Use Isotope Pattern in Precursor Reevaluation | True |
|  |  | Lower RT Limit | 0 |
|  | Spectrum Properties Filter  Scan Event Filters | Upper RT Limit | 0 |
|  |  | First Scan | 0 |
|  |  | Last Scan | 0 |
|  |  | Ignore Specified Scans | None |
|  |  | Lowest Charge State | 0 |
|  |  | Highest Charge State | 0 |
|  |  | Min. Precursor Mass | 350 Da |
|  |  | Max. Precursor Mass | 6,500 Da |
|  |  | Total Intensity Threshold | 0 |
|  |  | Minimum Peak Count | 1 |
|  |  | Mass Analyzer | Any |
|  | Scan Event Filters  Peak Filters | MS Order | Is Not MS1 |
|  |  | Activation Type | Any Activation Type |
|  |  | Min. Collision Energy | 0 |
|  |  | Max. Collision Energy | 1.000 |
|  |  | Scan Type | Is Full |
|  |  | Polarity Mode | Any |
|  |  | S/N Threshold (FT-only) | 1,5 |
|  | Replacements for Unrecognized Properties | Unrecognized Charge Replacements | Automatic |
|  | Replacements for Unrecognized Properties  Precursor Pattern Extraction | Unrecognized Mass Analyzer Replacements | Ion Trap (ITMS) |
|  |  | Unrecognized MS Order Replacements | MS2 |
|  |  | Unrecognized Activation Type Replacements | CID (Collision Induced Dissociation) |
|  |  | Unrecognized Polarity Replacements | Positive |
|  |  | Unrecognized MS Resolution@200 Replacements | 60.000 |
|  |  | Unrecognized MSn Resolution@200 Replacements | 30.000 |
|  |  | Precursor Clipping Range Before | 2.5 Da |
|  | Precursor Pattern Extraction  Input Data | Precursor Clipping Range After | 5.5 Da |
|  |  | Protein Database | Mouse_C57BL6J_Reference_Proteome_Canoncial_2022.fasta |
| Sequest HT  Perculator | Input Data  Tolerances | Enzyme Name | Trypsin (Full) |
|  |  | Max. Missed Cleavage Sites | 3 |
|  |  | Min. Peptide Length | 6 |
|  |  | Max. Peptide Length | 144 |
|  |  | Max. Number of Peptides Reported | 10 |
|  |  | Precursor Mass Tolerance | 10 ppm |
|  | Tolerances  Spectrum Matching |  | 0.02 Da |
|  |  | Use Average Precursor Mass | False |
|  |  | Use Average Fragment Mass | False |
|  |  | Use Neutral Loss a Ions | True |
|  | Spectrum Matching  Dynamic Modifications | Use Neutral Loss b Ions | True |
|  |  | Use Neutral Loss y Ions | True |
|  |  | Use Flanking Ions | True |
|  |  | Weight of a Ions | 0 |
|  |  | Weight of b Ions | 1 |
|  |  | Weight of c Ions | 0 |
|  |  | Weight of x Ions | 0 |
|  |  | Weight of y Ions | 1 |
|  |  | Weight of z Ions | 0 |
|  |  | Max. Equal Modifications Per Peptide | 3 |
|  | Dynamic Modifications  Dynamic Modification (Peptide Terminus) | Max. Dynamic Modifications Per Peptide | 4 |
|  |  | Dynamic Modification | Oxidation / +15.995 Da (M) |
|  |  | N-Terminal Modification | None |
|  | Dynamic Modification (Peptide Terminus)  Dynamic Modification (Protein Terminus) | C-Terminal Modification | None |
|  |  | N-Terminal Modification | None |
|  | Dynamic Modification (Protein Terminus)  Static Modifications | C-Terminal Modification | None |
|  |  | Static Modification | Carbamidomethyl / +57.021 Da (C) |
|  | Static Modifications | Static Modification | None |
|  |  | Peptide N-Terminus | None |
|  |  | Validation based on | q-Value |
| Perculator | Input Data | Maximum Delta Cn | 0,05 |
|  | Input Data  Decoy Database Search | Maximum Rank | 0 |
|  |  | Target FDR (Strict) | 0,01 |
|  | Decoy Database Search | Target FDR (Relaxed) | 0,05 |
|  |  | Validation based on | q-Value |

**Supplementary Table S4. Proteome Discoverer settings-consensus step.**

| MSF Files | Storage Settings | Spectra to Store | Identified or quantified |
| --- | --- | --- | --- |
|  |  | Feature Traces to Store | All |
|  |  | File Limit for Automatic Merge | 10 |
|  | Merging of Identified Peptide and Proteins | Merge Mode | Globally by Search Engine Type |
|  | FASTA Title Line Display | Reported FASTA Title Lines | Best match |
|  |  | Title Line Rule | Standard |
|  |  | Preferred Accession |  |
|  |  | Preferred Taxonomy |  |
|  |  | Avoid Expressions |  |
|  | PSM Filters | Maximum Delta Cn | 0,05 |
|  |  | Maximum Rank | 0 |
|  |  | Maximum Delta Mass | 0 ppm |
|  |  | Score |  |
|  |  | Threshold | 0 |
| PSM Grouper | Peptide Group Modifications | Site Probability Threshold | 75 |
| Peptide Validator | General Validation Settings | Validation Mode | Automatic (Control Peptide-Level Error Rate if Possible) |
|  |  | Target FDR (Strict) for PSMs | 0,01 |
|  |  | Target FDR (Relaxed) for PSMs | 0,05 |
|  |  | Target FDR (Strict) for Peptides | 0,01 |
|  |  | Target FDR (Relaxed) for Peptides | 0,05 |
|  | Specific Validation Settings | Validation Based On | q-Value |
|  |  | Target/Decoy Selection for PSM Level FDR | Automatic |
|  |  | Reset Confidence for Nodes without Decoy | False |
| Peptide and Protein Filter | Peptide Filters | Peptide Confidence at Least | High |
|  |  | Keep Lower Confident PSMs | False |
|  |  | Minimum Peptide Length | 6 |
|  |  | Remove Peptides Without Protein Reference | False |
|  | Protein Filter | Minimum Number of Peptide Sequences | 6 |
|  |  | Count Only Rank 1 Peptides | False |
|  |  | Count Peptides Only for Top Scored Protein | False |
| Protein Scorer |  |  |  |
| Protein FDR Validator | Confidence Threshold | Target FDR (Strict) | 0,01 |
|  |  | Target FDR (Relaxed) | 0,05 |
| Protein Grouping | Protein Grouping | Apply Strict Parsimony Principle | True |
| Peptide in Protein Annotation | Flanking Residues | Annotate Flanking Residue of the Peptide | True |
|  |  | Number Ranking Residues in Connection Tabs | 1 |
|  | Modifications in Peptide | Protein Modifications Reported | Only for Master Proteins |
|  | Modifications in Protein | Modifications Sites Reported | All And Specific |
|  |  | Minimum PSM Confidence | High |
|  |  | Report Only OTMs | TRUE |
|  |  | N-Terminal Modification | None |
|  |  | C-Terminal Modification | None |
|  | Positions in Protein | Protein Positions for Peptides | Only for Master Proteins |
